# Medication Intake Is Associated with Lower Plasma Carotenoids and Higher Fat-Soluble Vitamins in the Cross-Sectional MARK-AGE Study in Older Individuals

**DOI:** 10.3390/jcm9072072

**Published:** 2020-07-01

**Authors:** Daniela Weber, Bastian Kochlik, Wolfgang Stuetz, Martijn E. T. Dollé, Eugène H. J. M. Jansen, Beatrix Grubeck-Loebenstein, Florence Debacq-Chainiaux, Jürgen Bernhardt, Efstathios S. Gonos, Miriam Capri, Claudio Franceschi, Ewa Sikora, María Moreno-Villanueva, Alexander Bürkle, Tilman Grune

**Affiliations:** 1 Department of Molecular Toxicology, German Institute of Human Nutrition Potsdam-Rehbruecke (DIfE), 14558 Nuthetal, Germany; bastian.kochlik@dife.de (B.K.); scientific.director@dife.de (T.G.); 2 Institute of Nutritional Sciences (140), University of Hohenheim, 70599 Stuttgart, Germany; wolfgang.stuetz@uni-hohenheim.de; 3 Centre for Health Protection, National Institute for Public Health and the Environment (RIVM), 3720BA Bilthoven, The Netherlands; Martijn.Dolle@rivm.nl (M.E.T.D.); eugene.jansen@rivm.nl (E.H.J.M.J.); 4 Research Institute for Biomedical Aging Research, University of Innsbruck, 6020 Innsbruck, Austria; beatrix.grubeck@uibk.ac.at; 5 URBC-NARILIS, NAmur Research Institute for LIfe Sciences (NARILIS), University of Namur, 5000 Namur, Belgium; florence.chainiaux@unamur.be; 6 BioTeSys GmbH, 73728 Esslingen, Germany; j.bernhardt@biotesys.de; 7 Institute of Biology, Medicinal Chemistry and Biotechnology, National Hellenic Research Foundation, 116 34 Athina, Greece; sgonos@eie.gr; 8 CIG-Interdepartmental Center “L.Galvani”, DIMES, Department of Experimental, Diagnostic and Specialty Medicine, ALMA MATER STUDIORUM, University of Bologna, 40126 Bologna, Italy; miriam.capri@unibo.it (M.C.); claudio.franceschi@unibo.it (C.F.); 9 Department of Applied Mathematics of the Institute of ITMM, National Research Lobachevsky State University of Nizhny Novgorod, 603105 Nizhny Novgorod, Russia; 10 Laboratory of the Molecular Bases of Ageing, Nencki Institute of Experimental Biology, Polish Academy of Sciences, 02-093 Warsaw, Poland; e.sikora@nencki.gov.pl; 11 Molecular Toxicology Group, University of Konstanz, 78457 Konstanz, Germany; maria.moreno-villanueva@uni-konstanz.de (M.M.-V.); alexander.buerkle@uni-konstanz.de (A.B.); 12 Human Performance Research Centre, Department of Sport Science, Box 30, University of Konstanz, 78457 Konstanz, Germany

**Keywords:** medication intake, medical drugs, micronutrients, biomarkers, nutrition, age

## Abstract

The regular use of medication may interfere with micronutrient metabolism on several levels, such as absorption, turnover rate, and tissue distribution, and this might be amplified during aging. This study evaluates the impact of self-reported medication intake on plasma micronutrients in the MARK-AGE Project, a cross-sectional observational study in 2217 subjects (age- and sex-stratified) aged 35–75 years from six European countries that were grouped according to age. Polypharmacy as possible determinant of micronutrient concentrations was assessed using multiple linear regression models adjusted for age-group, dietary fruit, vegetables, and juice intake, and other confounders. Younger participants reported taking fewer drugs than older participants. Inverse associations between medication intake and lutein (−3.31% difference per increase in medication group), β-carotene (−11.44%), α-carotene (−8.50%) and positive associations with retinol (+2.26%), α-tocopherol/cholesterol (+2.89%) and γ-tocopherol/cholesterol (+1.36%) occurred in multiple adjusted regression models. Combined usage of a higher number of medical drugs was associated with poorer status of carotenoids on the one hand and higher plasma concentrations of retinol, α- and γ-tocopherol on the other hand. Our results raise concerns regarding the safety of drug combinations via the significant and surprisingly multifaceted disturbance of the concentrations of relevant micronutrients.

## 1. Introduction

While the mean life expectancy continues to rise, this is seemingly not accompanied by an increase in health span. There is intense scientific interest in finding strategies to enable ‘dynamic aging’, which means to increase health span by reducing the time period during which a person is ill or disabled. One strategy could be an optimized nutrition, since diet may be a safe, practical, realistic and cheap intervention to increase health span, as shown by protective effects of higher plasma concentrations and dietary intake of lipid-soluble micronutrients against age-related diseases [1,2,3,4,5,6,7]. In a recent study on European subjects (35–75 years) from the general population, we observed that age was negatively associated with concentrations of some fat-soluble micronutrients, even after adjusting for season, anthropometric data, cholesterol and dietary intake [8,9] and confirmed these findings in a German cohort [10]. Therefore, we concluded that age was one main contributor to micronutrient status in these cohorts.

The concentration of a certain micronutrient in plasma can accurately reflect recent or even long-term dietary habits, e.g., over a time period of 3–4 years in the case of the tomato-derived carotenoid lycopene [11]. However, since there is a discrepancy between micronutrient intake and plasma concentrations, there must be confounders other than the inaccuracy of dietary assessment or issues with the analytical methods used. One confounder could be the effect of medication, and further, the underlying diseases for which medication is used.

However, the impact of medication intake on plasma micronutrient concentrations has been scarcely studied and previous studies focused mainly on the association between polypharmacy and general nutritional status—but not single micronutrients—in older individuals (>65 years), in institutionalized persons or in hospitalized patients [12,13,14,15]. To the best of our knowledge, most data on the general frequency of medication intake originate only from older populations [16]. Furthermore, most publications examined only the effects of one specific drug on one specific micronutrient in one specific disease [17]. Samaras et al. found that proton pump inhibitors were associated with reduced absorption of vitamin C, B12 and iron, acetylsalicylic acid was associated with reduced absorption of vitamin C, and statin treatment was related to lower vitamin D, folate and coenzyme Q concentrations. However, it deserves attention that not only old persons consume multiple drugs and that drugs can affect several micronutrients simultaneously. In this study, we adhere to the definition of Fulton et al. of polypharmacy as the use of multiple drugs by a single person [18].

The regular use of medication may interfere with micronutrient metabolism on several concentrations such as absorption (competition, inhibition), excretion, turnover rate, or tissue distribution of specific nutrients [19,20], as well as on appetite, and these effects may be aggravated during aging. Thus, plasma micronutrient concentrations may be influenced due to the aforementioned reasons. Aging itself possibly alters metabolism of both nutrients and drugs, most often apparent as a slowdown [15]. Furthermore, there might be general age-related changes in bioavailability and metabolism of nutrients and drugs that are not related to one other.

Therefore, the aim of the present work was to evaluate the associations of self-reported medication with plasma micronutrient concentrations in a well-characterized age and sex-stratified European study population of subjects from the general population aged 35–75 years from the MARK-AGE Project [21,22,23,24].

## 2. Subjects and Methods

The study participants (total *n* = 2217) were from Austria (18.0%; *n* = 399), Belgium (11.8%; *n* = 261), Germany (16.1%; *n* = 357), Greece (17.9%; *n* = 397), Italy (18.0%; *n* = 398) and Poland (18.3%; *n* = 405), and belonged to the group of randomly recruited age-stratified subjects from the general population (RASIG) of the MARK-AGE study [21,22,23,24]. Individuals who reported seropositivity for HIV or hepatitis (hepatitis B or C), who tested positive for hepatitis B or C, were treated for cancer, or received glucocorticoids were excluded from the study [21,22,23,24]. 

### 2.1. Data Assessment and Biomarker Measurement

Recruitment of participants, study outline, assessment of anthropometrics and questionnaires have been described in detail elsewhere [8,9,21,22,23,24]. Participants were asked to report the use of prescribed medicine by questionnaire as follows: “Do you use any prescribed medicine on a regular basis”, with check boxes for “Yes” and “No”, and if “Yes” to fill in the “Name of medicine” and “For which disease?” ATC codes were filled out by interviewers when available/applicable. The participants could report up to 12 different drugs; however, the maximum number of drugs reported in this study was ten. We did not discriminate between the type of drug or the disease treated with the different drugs. Participants were asked to rate their physical health using a standard 5-point scale with the responses excellent, very good, good, fair, or poor [25,26]. The measurement of micronutrients (ascorbic acid, carotenoids, tocopherols, and retinol) by high-performance liquid chromatography and cholesterol using a clinical autoanalyzer (Beckman LX-20) has been described elsewhere in detail [8,9].

### 2.2. Ethics

Ethical approval for the study was given by the Local Research Ethics Committees for each of the recruiting centers. The MARK-AGE Project has retrospectively been registered at the German Clinical Trials Register (DRKS00007713). All participants gave written consent to participate in accordance with the Helsinki Declaration of 1975, as revised in 1983.

### 2.3. Statistical Analyses

Demographic characteristics were described using means and SD (standard deviations) for continuous variables (age, weight, height, BMI) and frequencies (%) for categorical variables (sex, smoking status, age-groups, and country). According to the subjects’ self-reported regular use of prescribed multiple drugs (“polypharmacy”), they were categorized into four groups: no use of medication (“no meds”), use of 1–2 medications regularly (“1–2 meds”), use of 3–4 medications regularly (“3–4 meds”), and use of five or more medications regularly (“≥5 meds”) (for frequencies see Table 1). Differences in characteristics between medication groups were determined by one-way ANOVA (continuous variables) and Pearson’s chi-squared test (prevalence). Data on plasma micronutrients were transformed to achieve normal distribution using square root (SR) or logarithmic (LN) transformation as appropriate and are described by geometric means with 95% confidence intervals (CI). Possible interactions of medication intake (Med-Group) and age (Age-Group) were examined for all biomarkers with the following model (Biomarker = a + b × Med-Group + c × Age-Group + d × (Med-Group × Age-Group)). Multiple linear regression analysis on associations of medication on biomarkers’ were adjusted for the term (Med-Group × Age-Group) for those biomarkers for which the interaction term was significant (*p* < 0.05).

Participants were categorized according to age into four age-groups with ten-year age-intervals (35–44 years, 45–54 years, 55–64 years, 65–75 years).

Polypharmacy as a potential determinant (4 groups) of biomarker concentrations was assessed using general linear models adjusted for age-group, season of blood collection, country, sex, BMI, smoking status, dietary intake (fruit, vegetables, and juice), and use of vitamin supplements, alcoholic drinks per month (beer, wine, other) and education (8 categories); partial Eta squared (ηp2) was applied as a measure of effect size. Regression coefficient B is used to show the increase/decrease in the respective micronutrient for linear regression models, and differences in biomarker concentrations were calculated as percentage (%) of the geometric means.

Statistically significant differences were considered to be present at *p* < 0.05. The greatest statistically significant differences of biomarkers with higher medication intake are presented as box plots. All statistical analyses were carried out using SPSS software (SPSS Inc., Chicago, IL, USA; Version 20). Microsoft PowerPoint was additionally used for figure preparation.

## 3. Results

A total of 2217 subjects from six European countries with a mean age of 55 years (range 35–75) were studied for medication intake and its association with plasma micronutrient concentrations.

The evaluation of participants’ characteristics regarding frequencies of self-reported medication intake is shown in Table 1. We found that 47.7% of participants reported taking “no meds” and 21.9% stated taking at least three meds. The participants consuming “no meds” were significantly younger than those consuming one or more regular medications; similarly, the subjects consuming “1–2 meds” were significantly younger than those consuming “3–4 meds” and “≥5 meds”, respectively. Sex, BMI, self-reported health status, and smoking were also associated with medication intake as shown in Table 1. The frequencies of participants reporting no use of regular medication were distributed equally among the six countries (17.4–20.1%, see Table 1), except for Belgium, with only 6.5% in the “no meds” group. Among those participants reporting taking “≥5 meds”, Belgium and Poland had the highest prevalence: 25.5% and 24.5%, respectively, while only 6.5% of participants in that group were residing in Germany.

**Figure 1:**
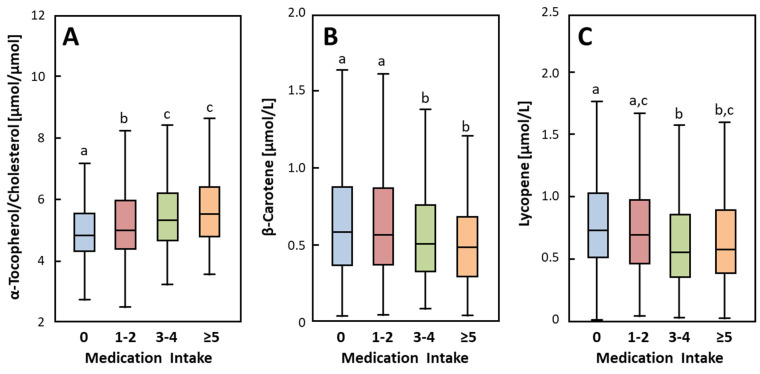
Plasma α-tocopherol/cholesterol (**A**), β-carotene (**B**) and lycopene (**C**) by frequency of self-reported medication intake. Extreme values are included in analyses but not shown in the figure. Box-plots sharing a common superscript letter are not significantly different (*p* < 0.05), as analyzed by one-way ANOVA with Tukey’s Post Hoc test. No medication (*n* = 1029); 1–2 regular meds (*n* = 640); 3–4 regular meds (*n* = 259); ≥5 regular meds (*n* = 190).

Table 2 shows the geometric means and 95% CIs for all plasma micronutrients separated by medication intake. Interestingly, we observed significantly higher concentrations of retinol, α- and γ-tocopherol, γ-tocopherol/cholesterol, and α-tocopherol/cholesterol in the “≥5 meds” group compared to the “no meds” and “1–2 meds” groups (Table 2 and Figure 1). For α-tocopherol, both the “1–2 meds” and “3–4 meds” groups had significantly higher concentrations than the “no meds” group (Table 2). In contrast, the concentrations of zeaxanthin, α-carotene, β-carotene and lycopene were significantly lower in the “≥5 meds” group compared to the “no meds” group (Table 2 and Figure 1). There were no differences in ascorbic acid, lutein, or β-cryptoxanthin concentrations between different medication groups.

The associations of plasma micronutrients with medication intake were assessed by linear regression analyses (Table 3). Retinol, γ-tocopherol, γ- and α-tocopherol/cholesterol were positively associated with medication intake, and these associations remained after adjusting for co-factors and covariates, except for γ-tocopherol. The positive association resulted in a higher concentration of retinol, γ- and α-tocopherol/cholesterol by 2.26%, 1.36% and 2.89% respectively, per increase in medication group, after adjusting for country, season, age-groups, sex, BMI, smoking status, frequency of dietary habits (fruit, vegetables, and juice per week), use of vitamin supplements, alcohol and education. α-Tocopherol was positively and inversely associated with medication intake in the unadjusted and in the adjusted model, respectively. γ-Tocopherol was positively associated with medication intake only in the unadjusted model. Lutein, which was not different between the individual medication groups, showed a significant inverse association with medication intake only in adjusted models. Ascorbic acid and β-cryptoxanthin were the only micronutrients not associated with medication intake either in the unadjusted or adjusted models. Supplemental Table S1 shows the associations of those micronutrients in the youngest and oldest age-groups after adjusting for covariables that did not show significant associations with medication intake.

All other micronutrients were inversely associated with medication intake. These inverse associations remained significant after adjusting the linear regression models, except for zeaxanthin, lycopene, and γ-tocopherol. We observed inverse associations between medication intake and lutein (−3.31% per increase in medication group), β-carotene (−11.44% per increase in medication group) and α-carotene (−8.50% per increase in medication group) in the adjusted models.

When distinguishing between a “young group (35–44 years)” and an “old group (65–75 years)”, results show significant associations of micronutrients with medication intake primarily in the “old group” (Table 4). Retinol and α-tocopherol/cholesterol ratio were positively associated, and zeaxanthin, β-carotene and α-tocopherol were negatively associated, with medication intake in the “old group” in both the unadjusted and the adjusted model. In the “young group”, only retinol and α-tocopherol (positively; adjusted model) and α-tocopherol/cholesterol (positively; unadjusted and adjusted model) were associated with medication intake. The frequencies of vitamin supplement intake, servings of juice, vegetables, fruits, French fries and meat are shown in Appendix A Table A1. The highest frequency of servings (≥7 servings/week) for vegetables, fruits and vitamin supplements and the lowest frequency for glasses of juice were observed in those participants taking ≥5 meds.

## 4. Discussion

Older people are frequent users of a wide range of medication, many of which are being used to treat chronic, age-related diseases. It is of importance to note that almost any kind of medication can potentially affect normal metabolism in many ways, most of which are poorly understood. To our knowledge, this is the first study evaluating the association of medication intake and plasma micronutrients in a large age-stratified cohort of subjects over a broad age range of 40 years starting at the age of 35 years.

The recent literature regarding medication intake focuses primarily on elderly (>65 years), institutionalized or hospitalized patients [12,13,14,15,16], and only a few publications included measures of micronutrients [12,14]. Bates et al. showed that 78% of British elderly (>65 years) living in the community and 93% of those in institutions were using medication. In our study, the frequency of 65-to-75-year-old subjects consuming ≥3 medications was 52.3%. Similarly, Heuberger et al. showed that 51.1% of participants >65 years were taking ≥5 medications [15]. Herr et al. reported that among 2350 older subjects (mean age 83.3 ± 7.5 years), 53.6% of the subjects received 5–9 drugs per day and 13.8% received ≥10 drugs per day [16]. We were able to demonstrate that the prevalence of participants taking at least one drug regularly increases from 26.4% (35–44 years) to 38.8% (45–54 years) to 61.9% (55–64 years) to 77.3% (65–75 years), and research in the younger age-groups is clearly scarce.

We observed lower concentrations of carotenoids (except β-cryptoxanthin and lycopene) with an increasing amount of medication intake and that this association is independent of confounders. After adjustment, the inverse association was strongest for β-carotene and α-carotene.

Remarkably, in our previous publication [9], lycopene, α-carotene, and β-cryptoxanthin were those carotenoids showing the strongest association with age, but in the current study there was no association between lycopene or β-cryptoxanthin with medication intake although age and medication intake were strongly correlated (*r* = 0.452, *p* < 0.001). This could imply that aging-related processes, such as metabolic alterations in the gastrointestinal tract or liver, and not the medication intake *per se* might be responsible for our controversial findings of an association with age but not with medication intake.

Bates et al. observed an inverse association between central nervous system medication and plasma lutein [12]. In another cross-sectional study with 1100 participants >65 years, the intake of fat-soluble vitamins and carotenoids was also lower in those with increasing medication use [15]. It is known that fruit and vegetable consumption among elderly persons is usually lower than recommended [27]. However, in our models adjusted for fruit and vegetable intake, the inverse association between medication intake and micronutrients remained significant. Thus, a poor intake of fruits and vegetables is likely not the only reason for the lower concentrations of carotenoids. Plasma ascorbic acid was not associated with medication intake, which is in accordance with another publication, focusing on a population >65 years [12].

There is one study that also investigated blood micronutrients and their association with medication intake [14]. The authors analyzed plasma concentrations of vitamins A, D, E, K, and C as well as functional parameters (related enzyme activity) of vitamins B_1_, B_2_, and B_6_ in 102 non-institutionalized subjects aged 70–90 years. They showed that those participants taking ≥3 drugs/day had significantly lower concentrations of vitamin D, K, B_6_, and folate, but there was no difference concerning vitamins A and E as we have observed. Unfortunately, there was no younger group.

In agreement with our findings, other authors also observed higher retinol and α-tocopherol concentrations in medication users compared to non-users [12] and in those taking ≥3 drugs compared to those taking ≤3 drugs [14].

Bates et al. observed a positive association between retinol and anti-hypertensives, gastrointestinal, central nervous system medication, and corticosteroids [12]. In our study, retinol correlated positively with diastolic and systolic blood pressure (*r* = 0.141 and *r* = 0.151, respectively; *p* < 0.001, *n* = 2216; not shown). High retinol concentrations may serve as an indicator for blood pressure or blood-pressure medication, or renal function impairment. Chen et al. showed that hypertensive participants (*n* = 4860) had significantly higher vitamin A, E, C, and β-carotene concentrations than normotensives (*n* = 10,457) [28]. Danquah et al. demonstrated that increased retinol in individuals with hypertension was mainly attributed to impaired kidney function in a Ghanaian population [29].

The hypothesis that medication may alter vitamin status by affecting metabolism is not new [12]. This could happen by affecting the availability, turnover, excretion or tissue storage of vitamins and of their respective carrier proteins. However, causal effects can be manifold and are difficult to interpret based on data of our study. For instance, a generally poor health status could lead to increased use of medication. It has been previously shown that health status appeared worse in older adults taking multiple medications than in older adults taking less medication [13]. Furthermore, it remains unclear whether an unhealthy lifestyle/diet with low micronutrient concentrations might be the reason for diseases or it is only associated with the occurrence of diseases, which in turn results in the intake of medications. Additionally, the question of whether medication inhibits the absorption of micronutrients, thus leading to malnutrition/undernourishment or vice versa, remains unanswered. Possible mechanisms involved in this drug–nutrition interaction and their accompanying adverse health effect seem to be mediated by cytochrome P450 dependent monooxygenases (CYP). Many types of drugs are metabolized by CYP and different drugs can react on CYP. Additionally, CYP can be involved in the metabolism of fat-soluble vitamins and can be stimulated or inhibited by foods/nutrition. Further research should be carried out regarding this field due to its relation to aging, diet and drugs.

All micronutrients analyzed in the present study display antioxidant properties, although retinol is not an accepted in vivo antioxidant. The water-soluble antioxidant ascorbic acid (vitamin C) was not associated with medication intake. This may be linked to the facts that ascorbic acid concentration fluctuates due to recent exposure [30], is subject to oxidation, and requires specific treatment for storage. In addition, it is neither stored nor transported in lipo- or plasma proteins like the carotenoids. These reasons may have contributed to our findings. In contrast to ascorbic acid, the lipophilic carotenoids reflect habitual dietary fruit and vegetable intake and the storage stability has been shown over a period of up to ten years and more. When comparing redox biomarkers among the four different medication groups, we observed higher malondialdehyde and lower total glutathione in the “≥5 meds” compared to “no meds” group, i.e., higher oxidative stress. This is supported by the observation of lower antioxidant concentrations of lycopene, α-, β- carotene and zeaxanthin in these participants.

There are some limitations of the work presented. The study was cross-sectional in nature, and therefore no causal relationships can be identified. Dietary analyses were completed at one time point only and may not have been representative for individual’s nutrient intake over time. Our observations might only reflect a low intake of fruits and vegetables. Unfortunately, we can only adjust our models for frequency of dietary intake because the information about nutritional data was limited. We did not adjust for socio-economic status and income, which are associated with health status. Furthermore, we did not discriminate between types of medication and dosages; subjects only stated “regular use”, which does not indicate the actual frequency of use. In our study, the reasons for drug intake were manifold but we did not further focus on different diseases and medications. Furthermore, there may be problems associated with self-reported medication intake, shown by a poor overlap of hospital medication records and self-reported medication. Lee et al. showed that 94% of personal medication lists had at least one discrepancy with hospital medication lists [31]. Therefore, the results obtained from this study may not be generalizable to other study populations.

Our study consisted of a large sample size, providing enormous statistical power to elucidate the association between plasma micronutrients and medication intake. Additionally, subjects from different European countries were analyzed, thus our results reflect a broad range of society and different lifestyles. Furthermore, high-quality blood analyses were performed, by only one laboratory, thus providing robust results without operator bias.

## 5. Conclusions

The main conclusions from the present study are that the use of medication is widespread among middle-aged and older people in six European countries and that this medication intake is associated with plasma micronutrient concentrations, even after adjusting for confounders. A higher number of drugs used by participants were associated with a poorer status of several carotenoids and with higher concentrations of the fat-soluble vitamins retinol and cholesterol-adjusted α- and γ-tocopherol.

An unknown (but probably not a minor) share of health care costs might result from overprescribing of drugs in addition to a non-optimal micronutrient status.

Our results raise concerns regarding the safety of medical drug combinations via the significant and surprisingly multifaceted disturbance of the concentrations of relevant micronutrients.

Additionally, in view of rising medication prescriptions and potentially inappropriate medications, reduction in medication intake (“deprescribing”) may be an alternative approach, beside an improved dietary intake, to contribute to a normal micronutrient metabolism and status.

The biological and clinical relevance remain vague at the present time; however, it is obvious, that future research in this field may lead to changes in clinical practice in terms of micronutrient status monitoring and supplementation of persons under polymedication, which is often neglected not only in hospitalized but also in free living individuals.

In future studies, functional parameters of micronutrients should be included such as enzyme activity as readout for B-vitamins, bone density measurements, hormone status, redox biomarkers as a proxy for antioxidant function or for instance neutrophil motility as a novel functional marker of vitamin C [32]. These parameters would be especially meaningful in longitudinal or intervention studies.

Our study reveals novel insights into a hardly-researched field on the verge between medical and nutritional science, combining both fields and pointing out possible synergistic or antagonistic effects, in a population that is neither diseased nor completely disease free. We believe our findings are a meaningful contribution that hopefully lead to more research on this topic, especially by including information of medication intake in younger study groups.

Further studies are needed to explain these associations mechanistically and evaluate which associations may be the result either of specific disease, or of metabolic effects of certain medications, which could lead to an adjustment of dietary recommendations for regular users of multiple medications.

## Figures and Tables

**Table 1:** Characteristics of the study population by self-reported medication intake.

	All	No Regular Meds	1–2 Regular Meds	3–4 Regular Meds	≥5 Regular Meds	*p*
**%** (***n***)	100 (2217)	47.7 (1058)	30.4 (675)	12.8 (284)	9.0 (200)	
**Age** (**years**) **^1^**	55.3 ± 11.4	50.6 ± 10.4 ^a^	56.3 ± 10.9 ^b^	63.4 ± 8.6 ^c^	65.0 ± 7.1 ^c^	<0.001
35–44 years (%, (*n*))	21.9 (485)	73.6 (357)	24.1 (117)	2.1 (10)	0.2 (1)	<0.001
45–54 years (%, (*n*))	25.3 (562)	61.2 (344)	31.0 (174)	5.3 (30)	2.5 (14)	
55–64 years (%, (*n*))	26.7 (593)	38.1 (226)	33.6 (199)	16.0 (95)	12.3 (73)	
65–75 years (%, (*n*))	26.0 (577)	22.7 (131)	32.1 (185)	25.8 (149)	19.4 (112)	
**Sex, Male** (**%**, (*n*))	49.3 (1092)	55.0 (582)	42.8 (289)	47.9 (136)	42.5 (85)	<0.001
**Height** (**cm**) **^1^**	169.1 ± 9.5	170.9 ± 9.4 ^a^	168.3 ± 9.4 ^b^	166.6 ± 9.4 ^c^	166.4 ± 9.3 ^b,c^	<0.001
**Weight** (**kg**) **^1^**	75.1 ± 15.0	75.2 ± 14.9 ^a,b^	73.6 ± 14.6 ^a^	76.5 ± 15.3 ^b^	77.4 ± 15.3 ^b^	0.003
**BMI** (**kg/m^2^**) **^1^**	26.2 ± 4.5	25.7 ± 4.4 ^a^	25.9 ± 4.4 ^a^	27.5 ± 4.5 ^b^	27.9 ± 4.6 ^b^	<0.001
<25 (%, (*n*))	44.9 (995)	50.0 (528)	47.1 (318)	31.7 (90)	29.5 (59)	<0.001
25–29.9 (%, (*n*))	38.0 (843)	36.3 (384)	37.3 (252)	44.0 (125)	41.0 (82)	
≥30 (%, (*n*))	17.1 (378)	13.7 (145)	15.6 (105)	24.3 (69)	29.5 (59)	
**Self-reported health status**						<0.001
excellent (%, (*n*))	11.5 (256)	15.5 (164)	11.0 (74)	3.9 (11)	3.5 (7)	
very good (%, (*n*))	37.8 (837)	45.8 (485)	36.0 (243)	27.1 (77)	16.0 (32)	
good (%, (*n*))	39.4 (873)	33.5 (354)	44.4 (300)	46.8 (133)	43.0 (86)	
fair (%, (*n*))	9.8 (218)	4.6 (49)	7.6 (51)	18.7 (53)	32.5 (65)	
poor (%, (*n*))	1.5 (33)	0.6 (6)	1.0 (7)	3.5 (10)	5.0 (10)	
**Smoker, current** (**%,** (***n***))	19.1 (424)	20.3 (215)	18.4 (124)	18.6 (53)	16.1 (32)	0.480
**Country**						
Austria (%, (*n*))	18.0 (399)	20.1 (213)	17.2 (116)	13.0 (37)	16.5 (33)	<0.001
Belgium (%, (*n*))	11.8 (261)	6.5 (69)	12.9 (87)	19.0 (54)	25.5 (51)	
Germany (%, (*n*))	16.1 (357)	18.5 (196)	17.3 (117)	10.9 (31)	6.5 (13)	
Greece (%, (*n*))	17.9 (397)	19.7 (208)	15.9 (107)	18.7 (53)	14.5 (29)	
Italy (%, (*n*))	18.0 (398)	17.4 (184)	21.5 (145)	15.5 (44)	12.5 (25)	
Poland (%, (*n*))	18.3 (405)	17.8 (188)	15.3 (103)	22.9 (65)	24.5 (49)	

^1^ Values are means ± S.D., *p*-value: one-way ANOVA (continuous variables) and Pearson’s chi-squared test (prevalence), *n* = 2217. ^a,b,c^ Means in a row without a common superscript letter differ statistically significantly (*p* < 0.05), as analyzed by one-way ANOVA with Tukey’s Post Hoc test.

**Table 2:** Biomarker concentrations by self-reported medication intake. ^1^

	No Medication (*n* = 1029)	1–2 Regular Meds (*n* = 640)	3–4 Regular Meds (*n* = 259)	≥5 Regular Meds (*n* = 190)	*p*
**Ascorbic Acid** (**mg/L**)	4.20 (4.01; 4.40)	4.46 (4.22; 4.72)	4.41 (4.01; 4.83)	3.84 (3.44; 4.26)	0.068
**Retinol** (**µmol/L**)	1.67 (1.64; 1.69) ^a^	1.70 (1.67; 1.74) ^a,b^	1.78 (1.72; 1.83) ^b,c^	1.84 (1.77; 1.91) ^c^	0.000
**Lutein** (**µmol/L**)	0.277 (0.268; 0.286)	0.278 (0.266; 0.291)	0.276 (0.258; 0.296)	0.259 (0.237; 0.282)	0.391
**Zeaxanthin** (**µmol/L**)	0.046 (0.045; 0.048) ^a^	0.045 (0.042; 0.047) ^a,b^	0.044 (0.040; 0.047) ^a,b^	0.039 (0.035; 0.043) ^b^	0.011
**β-Cryptoxanthin** (**µmol/L**)	0.200 (0.190; 0.211)	0.215 (0.201; 0.231)	0.198 (0.176; 0.224)	0.186 (0.163; 0.212)	0.176
**α-Carotene** (**µmol/L**)	0.145 (0.139; 0.153) ^a^	0.146 (0.137; 0.155) ^a^	0.116 (0. 107; 0.127) ^b^	0.121 (0.107; 0.137) ^b^	0.000
**β-Carotene** (**µmol/L**)	0.564 (0.541; 0.587) ^a^	0.555 (0.528; 0.583) ^a^	0.479 (0.443; 0.518) ^b^	0.459 (0.413; 0.511) ^b^	0.000
**Lycopene** (**µmol/L**)	0.748 (0.723; 0.773) ^a^	0.702 (0.673; 0.732) ^a,c^	0.582 (0.536; 0.629)^b^	0.621 (0.566; 0.678) ^b,c^	0.000
**α-Tocopherol** (**µmol/L**)	27.3 (26.9; 27.8) ^a^	28.3 (27.7; 28.8) ^b^	28.7 (27.8; 29.6) ^b^	28.3 (27.2; 29.5) ^a,b^	0.008
**γ-Tocopherol** (**µmol/L**)	1.27 (1.22; 1.31)	1.26 (1.20; 1.32)	1.36 (1.31; 1.48)	1.38 (1.28; 1.49)	0.018
**α-Tocopherol/Cholesterol** (**µmol/mmol**)	4.95 (4.89; 5.02) ^a^	5.15 (5.05; 5.24) ^b^	5.41 (5.27; 5.55) ^c^	5.64 (5.45; 5.83) ^c^	0.000
**γ-Tocopherol/Cholesterol** (**µmol/mmol**)	0.231 (0.223; 0.238) ^a^	0.232 (0.222; 0.243) ^b^	0.265 (0.258; 0.282) ^c^	0.277 (0.258; 0.298) ^c^	0.000
**Cholesterol** (**mmol/L**)	5.62 (5.56; 5.68) ^a^	5.62 (5.53; 5.70) ^a,b^	5.42 (5.29; 5.54) ^b^	5.14 (4.97; 5.30) ^c^	0.000

^1^ Data are geometric means (95% CI). ^a,b,c^ Geometric means in a row without a common superscript letter differ statistically significantly (*p* < 0.05), as analyzed by one-way ANOVA with Tukey’s Post Hoc test.

**Table 3:** Association of medication intake with biomarkers after adjusting for covariables.

	B	95% CI	(η^2^)	Difference ^3^ (Unit)	Difference ^4^ (%)	*p*
*(**SR**) **Ascorbic Acid** (**mg/L**)*						
Medication (4 Groups)	−0.008	(−0.042; 0.026)	0.000	−0.032	−0.75	0.657
Medication (4 Groups) adjusted ^1^	−0.016	(−0.044; 0.012)	0.001	−0.086	−2.02	0.276
*(**SR**) **Retinol** (**µmol/L**)*						
Medication (4 Groups)	0.021	(0.014; 0.029)	0.016	0.058	3.38	0.000
Medication (4 Groups) adjusted ^1^	0.017	(0.009; 0.024)	0.009	0.038	2.26	0.000
*(**SR**) **Lutein** (**µmol/L**)*						
Medication (4 Groups)	−0.004	(−0.010; 0.003)	0.001	−0.004	−1.44	0.241
Medication (4 Groups) adjusted ^1^	−0.007	(−0.014; −0.000)	0.002	−0.009	−3.31	0.037
*(**SR**) **Zeaxanthin** (**µmol/L**)*						
Medication (4 Groups)	−0.005	(−0.008; −0.002)	0.005	−0.002	−4.43	0.002
Medication (4 Groups) adjusted ^1,2^	0.004	(−0.007; 0.014)	0.000	−0.002	−4.49	0.468
*(**Ln**) **β-Cryptoxanthin** (**µmol/L**)*						
Medication (4 Groups)	−0.013	(−0.053; 0.026)	0.000	−0.003	−1.29	0.511
Medication (4 Groups) adjusted ^1^	−0.033	(−0.070; 0.004)	0.001	−0.008	−3.90	0.083
*(**Ln**) **α-Carotene** (**µmol/L**)*						
Medication (4 Groups)	−0.073	(−0.108; −0.038)	0.008	−0.009	−6.48	0.000
Medication (4 Groups) adjusted ^1^	−0.045	(−0.078; −0.012)	0.004	−0.012	−8.50	0.007
*(**Ln**) **β-Carotene** (**µmol/L**)*						
Medication (4 Groups)	−0.069	(−0.099; −0.040)	0.010	−0.033	−6.18	0.000
Medication (4 Groups) adjusted ^1^	−0.060	(−0.088; −0.032)	0.009	−0.062	−11.44	0.000
*(**SR**) **Lycopene** (**µmol/L**)*						
Medication (4 Groups)	−0.034	(−0.044; −0.023)	0.019	−0.052	−7.54	0.000
Medication (4 Groups) adjusted ^1^	−0.010	(−0.021; 0.001)	0.002	−0.025	−3.52	0.063
*(**Ln**) **α-Tocopherol** (**µmol/L**)*						
Medication (4 Groups)	0.017	(0.005; 0.028)	0.004	0.485	1.74	0.004
Medication (4 Groups) adjusted ^1,2^	0.053	(0.013; 0.092)	0.003	−1.282	−4.60	0.009
*(**Ln**) **γ-Tocopherol** (**µmol/L**)*						
Medication (4 Groups)	0.032	(0.008; 0.057)	0.003	0.044	3.41	0.010
Medication (4 Groups) adjusted ^1,2^	0.040	(−0.038; 0.118)	0.000	−0.023	−1.77	0.312
*(**Ln**) **α-Tocopherol/Cholesterol** (**µmol/mmol**)*						
Medication (4 Groups)	0.043	(0.034; 0.053)	0.038	0.238	4.63	0.000
Medication (4 Groups) adjusted ^1^	0.034	(0.024; 0.045)	0.020	0.148	2.89	0.000
*(**Ln**) **γ-Tocopherol/Cholesterol** (**µmol/mmol**)*						
Medication (4 Groups)	0.061	(0.037; 0.085)	0.012	0.016	6.78	0.000
Medication (4 Groups) adjusted ^1^	0.031	(0.006; 0.055)	0.003	0.003	1.36	0.013
***Cholesterol** (**mmol/L**)*						
Medication (4 Groups)	−0.135	(−0.181; −0.089)	0.016	−0.135	−2.43	0.000
Medication (4 Groups) adjusted ^1,2^	0.094	(−0.072; 0.260)	0.001	−0.094	−1.70	0.267

Multiple linear regression analysis with medication groups (*n* = 4) as covariate; (SR) square root transformed; (Ln) logarithmic transformed. Regression coefficient B represents the increase/decrease in the respective compound for each linear regression model. ^1^ Adjusted for age-groups (*n* = 4), sex, BMI, smoking status, frequency of dietary habits (fruit, vegetables, and juice per week), use of vitamin supplements, alcoholic drinks per month (beer, wine, other) and education as covariates, and country and season as co-factors. ^2^ Adjustment for interaction term [Medication Group × Age-Group]. ^3^ Mean differences per medication intake of back-transformed data (unit) considering the intercept of each model.^4^ Differences in biomarker concentrations as percentage (%) of the geometric means (Ascorbic Acid: 4.271 mg/L; Retinol: 1.705 µmol/L; Lutein: 0.276 µmol/L; Zeaxanthin: 0.045 µmol/L; β-Cryptoxanthin: 0.203 µmol/L; α-Carotene: 0.139 µmol/L; β-Carotene: 0.540 µmol/L; Lycopene: 0.701 µmol/L; α-Tocopherol: 27.9 µmol/L; γ-Tocopherol: 1.287 µmol/L; α-Tocopherol/Cholesterol: 5.127 µmol/mmol; γ-Tocopherol/Cholesterol: 0.239 µmol/mmol; Cholesterol: 5.55 mmol/L).

**Table 4:** Association of medication with biomarkers in youngest and oldest age-groups after adjusting for covariables.

	Age-Group: 35–44 Years					Age-Group: 65–75 Years				
	B (95% CI)	(η^2^)	Diff. ^3^ (Unit)	Diff. ^4^ (%)	*p*	B (95% CI)	(η^2^)	Diff. ^3^ (Unit)	Diff. ^4^ (%)	*p*
*(**SR**) **Retinol** (**µmol/L**)*										
Medication (4 Groups)	0.024 (−0.006; 0.054)	0.005	0.063	3.90	0.118	0.016 (0.003; 0.030)	0.010	0.041	2.37	0.019
Medication (4 Groups), adj. ^1^	0.032 (0.004; 0.060)	0.012	0.075	4.66	0.023	0.028 (0.014; 0.041)	0.033	0.079	4.55	0.000
*(**SR**) **Zeaxanthin** (**µmol/L**)*										
Medication (4 Groups)	−0.005 (−0.017; 0.006)	0.002	−0.002	−4.91	0.363	−0.012 (−0.018; −0.006)	0.032	−0.006	−13.97	0.000
Medication (4 Groups), adj. ^1,2^	−0.009 (−0.021; 0.003)	0.005	−0.002	−4.89	0.148	−0.008 (−0.013; −0.002)	0.014	−0.004	−8.85	0.008
*(**Ln**) **β*** ***-Carotene*** *(**µmol/L**)*										
Medication (4 Groups)	−0.032 (−0.148; 0.085)	0.001	−0.017	−2.96	0.592	−0.061 (−0.113; −0.009)	0.010	−0.040	−7.71	0.022
Medication (4 Groups), adj. ^1^	−0.035 (−0.140; 0.070)	0.001	−0.027	−4.92	0.511	−0.063 (−0.110; −0.017)	0.014	−0.058	−11.15	0.008
*(**Ln**) **α*** ***-Tocopherol*** *(**µmol/L**)*										
Medication (4 Groups)	0.037 (−0.008; 0.081)	0.006	1.009	3.97	0.107	−0.030 (−0.052; −0.007)	0.012	−0.971	−3.33	0.010
Medication (4 Groups), adj. ^1,2^	0.048 (0.002; 0.093)	0.010	0.789	4.89	0.039	−0.023 (−0.045; −0.001)	0.008	−0.827	−2.83	0.042
*(**Ln**) **α-Tocopherol/Cholesterol** (**µmol/mmol**)*										
Medication (4 Groups)	0.060 (0.023; 0.096)	0.022	0.332	6.83	0.002	0.029 (0.010; 0.048)	0.018	0.140	2.59	0.003
Medication (4 Groups), adj. ^1^	0.059 (0.020; 0.097)	0.021	0.324	6.65	0.003	0.039 (0.013; 0.058)	0.032	0.200	3.70	0.000
*(**Ln**) **Cholesterol** (**mmol/L**)*										
Medication (4 Groups)	−0.119 (−0.296; 0.058)	0.004	−0.119	−2.24	0.187	−0.300 (−0.389; −0.211)	0.078	−0.300	−5.43	0.000
Medication (4 Groups), adj. ^1,2^	−0.037 (−0.226; 0.153)	0.000	−0.037	−0.69	0.705	−0.290 (−0.382; −0.199)	0.076	−0.290	−5.25	0.000

Multiple linear regression with medication group (*n* = 4) as covariate; (SR) square root transformed; (Ln) logarithmic transformed. Regression coefficient B represents the increase/decrease in the respective compound for each multiple linear regression model. ^1^ Adjusted for age (linear), sex, BMI, smoking status, frequency of dietary habits (fruit, vegetables, and juice per week), use of vitamin supplements as covariates, alcoholic drinks per month (beer, wine, other) and education and country and season as co-factors. ^2^ Adjustment for interaction term [Medication Group × Age-Group]. ^3^ Mean differences per medication intake of back-transformed data (unit) considering the intercept of each model. ^4^ Differences in biomarker concentrations as percentage (%) of the geometric means (Retinol: 35–44 years: 1.606 µmol/L; 65–75 years: 1.748 µmol/L; Zeaxanthin: 35–44 years: 0.041 µmol/L; 65–75 years: 0.044 µmol/L; β-Carotene: 35–44 years: 0.557 µmol/L; 65–75 years: 0.520 µmol/L; α-Tocopherol: 35–44 years: 25.4 µmol/L; 65–75 years: 29.2 µmol/L; α-Tocopherol/Cholesterol: 35–44 years: 4.863 µmol/mmol; 65–75 years: 5.403 µmol/mmol; Cholesterol: 35–44 years: 5.326 mmol; 65–75 years: 5.528 mmol).

## Supplementary Materials

The following is available online at https://www.mdpi.com/2077-0383/9/7/2072/s1, Supplemental Table S1: Association of medication intake with biomarkers in the youngest and oldest age-groups after adjusting for covariables.

## Appendix A

**Table A1:** Frequencies of dietary intake by medication groups.

	Total (*n* = 2217)	No Medication (*n* = 1058)	1–2 Regular Meds (*n* = 675)	3–4 Regular Meds (*n* = 285)	≥5 Regular Meds (*n* = 199)	*p*-Value
**Vitamin supplements**, **%** (***n***)						<0.001
never	63.7 (1413)	67.1 (710)	61.9 (418)	58.6 (167)	59.3 (118)	
<1 serving per week	16.4 (364)	16.0 (169)	17.5 (118)	17.9 (51)	13.1 (26)	
1–6 servings per week	8.3 (183)	8.3 (88)	8.7 (59)	9.5 (27)	4.5 (9)	
≥7 servings per week	11.6 (257)	8.6 (91)	11.9 (80)	14.0 (40)	23.1 (46)	
**Glasses of juice**, **%** (***n***)						<0.001
never	26.6 (590)	21.6 (228)	28.6 (193)	31.9 (91)	39.2 (78)	
<1 serving per week	13.6 (302)	12.6 (133)	14.7 (99)	16.1 (46)	12.1 (24)	
1–6 servings per week	28.1 (624)	31.0 (328)	26.1 (176)	26.3 (75)	22.6 (45)	
≥7 servings per week	31.6 (701)	34.9 (369)	30.7 (207)	25.6 (73)	26.1 (52)	
**Vegetable servings**, **%** (***n***)						<0.001
never	0.1 (2)	0.0 (0)	0.3 (2)	0.0 (0)	0.0 (0)	
<1 serving per week	0.1 (2)	0.1 (1)	0.1 (1)	0.0 (0)	0.0 (0)	
1–6 servings per week	39.6 (878)	45.4 (480)	33.6 (227)	38.2 (109)	31.2 (62)	
≥7 servings per week	60.2 (1335)	54.5 (577)	65.9 (445)	61.8 (176)	68.8 (137)	
**Fruit servings**, **%** (***n***)						<0.001
never	0.6 (13)	0.6 (6)	0.4 (3)	0.0 (0)	2.0 (4)	
<1 serving per week	4.7 (104)	5.4 (57)	4.7 (32)	2.5 (7)	4.0 (8)	
1–6 servings per week	30.1 (668)	34.8 (368)	25.6 (173)	30.9 (88)	19.6 (39)	
≥7 servings per week	64.6 (1432)	59.3 (627)	69.2 (467)	66.7 (190)	74.4 (148)	
**Servings of French fries**, **%** (***n***)						0.045
never	26.7 (591)	22.8 (241)	30.1 (203)	29.8 (85)	31.2 (62)	
<1 serving per week	56.4 (1251)	60.2 (637)	53.3 (360)	52.6 (150)	52.3 (104)	
1–6 servings per week	16.1 (358)	16.3 (172)	15.9 (107)	16.5 (47)	16.1 (32)	
≥7 servings per week	0.8 (17)	0.8 (8)	0.7 (5)	1.1 (3)	0.5 (1)	
**Meat servings**, **%** (***n***)						0.123
never	1.2 (26)	1.8 (19)	0.7 (5)	0.4 (1)	0.5 (1)	
<1 serving per week	8.4 (186)	7.8 (83)	9.0 (61)	7.4 (21)	10.6 (21)	
1–6 servings per week	78.2 (1734)	78.4 (830)	79.4 (536)	77.9 (222)	73.4 (146)	
≥7 servings per week	12.2 (271)	11.9 (126)	10.8 (73)	14.4 (41)	15.6 (31)	

*p*-value determined by Pearson’s chi-squared test (prevalence).
