# Supplementary material for: Medication Intake Is Associated with Lower Plasma Carotenoids and Higher Fat-Soluble Vitamins in the Cross-Sectional MARK-AGE Study in Older Individuals
Source: J Clin Med. 2020 Jul 1;9(7):2072. doi: 10.3390/jcm9072072 (PMC7408954; doi:10.3390/jcm9072072)
Supplement: Supplementary file 1 [file jcm-09-02072-s001.pdf]

Supplemental Table S1. Association of medication intake with biomarkers in the youngest and oldest age groups after adjusting for covariables

	Age Group: 35 - 44 years					Age Group: 65 - 75 years				
	B (95% CI)	(η^2)	Difference [2] (unit)	Difference [3] (%)	*P*	B (95% CI)	(η^2)	Difference [2] (unit)	Difference [3] (%)	*P*
(SR) Ascorbic Acid (mg/L)										
Medication (4 Groups)	0.085 (-0.058; 0.228)	0.003	0.043	1.01	0.244	-0.038 (-0.088; 0.013)	0.003	-0.034	-7.92	0.142
Medication (4 Groups), adj. [1]	0.002 (-0.102; 0.106)	0.000	0.69	0.03	0.971	-0.004 (-0.042; 0.034)	0.000	-0.078	-1.81	0.832
(SR) Lutein (µmol/l)										
Medication (4 Groups)	-0.011 (-0.036; 0.013)	0.002	-0.012	-4.76	0.365	-0.009 (-0.021; 0.003)	0.004	-0.010	-3.64	0.147
Medication (4 Groups), adj. [1]	-0.018 (-0.041; 0.006)	0.005	-0.016	-6.40	0.140	-0.003 (-0.015; 0.009)	0.000	-0.004	-1.62	0.650
(Ln) α-Carotene (µmol/l)										
Medication (4 Groups)	0.093 (-0.060; 0.247)	0.003	0.011	7.23	0.232	-0.054 (-0.118; 0.011)	0.005	-0.008	-6.63	0.102
Medication (4 Groups), adj. [1]	-0.055 (-0.187; 0.078)	0.001	-0.013	-8.31	0.418	-0.067 (-0.125; -0.008)	0.010	-0.039	-31.42	***0.025***
(Ln) β-Cryptoxanthin (µmol/L)										
Medication (4 Groups)	-0.030 (-0.175; 0.116)	0.000	-0.005	-3.20	0.690	-0.047 (-0.109; 0.015)	0.003	-0.012	-5.58	0.138
Medication (4 Groups), adj. [1]	-0.089 (-0.224; 0.045)	0.004	-0.018	-10.66	0.191	0.012 (-0.038; 0.062)	0.000	0.015	6.65	0.641
(SR) Lycopene (µmol/l)										
Medication (4 Groups)	-0.040 (-0.079; -0.001)	0.008	-0.081	-9.99	***0.046***	-0.006 (-0.024; 0.013)	0.001	-0.008	-1.56	0.555
Medication (4 Groups), adj. [1]	-0.019 (-0.058; 0.020)	0.002	-0.047	-5.83	0.339	-0.006 (-0.024; 0.013)	0.001	-0.020	-3.74	0.553
(Ln) γ-Tocopherol (µmol/l)										
Medication (4 Groups)	0.017 (-0.089; 0.123)	0.000	0.018	1.62	0.753	-0.030 (-0.074; 0.014)	0.003	-0.045	-3.35	0.184
Medication (4 Groups), adj. [1]	-0.044 (-0.136; 0.048)	0.002	-0.015	-1.36	0.345	-0.022 (-0.067; 0.022)	0.002	-0.016	-1.21	0.326
(Ln) γ-Tocopherol/Cholesterol (µmol/mmol)										
Medication (4 Groups)	0.056 (-0.045; 0.161)	0.003	0.010	4.83	0.288	0.033 (-0.010; 0.077)	0.004	0.007	2.88	0.134
Medication (4 Groups), adj. [1]	-0.015 (-0.107; 0.078)	0.000	-0.001	-0.58	0.753	0.042 (0.000; 0.085)	0.008	0.004	1.53	0.052

Multiple linear regression with medication group (n = 4) as covariate; (SR) square root transformed; (Ln) logarithmic transformed. Regression coefficient B represents the increase/decrease in the respective compound for each multiple linear regression model.

[1] adjusted for age (linear), sex, BMI, smoking status, frequency of dietary habits (fruit, vegetables, and juice per week), and use of vitamin supplements as covariates, and country and season as co-factors.

[2] Mean differences per medication intake of back-transformed data (unit) considering the intercept of each model;

[3] Differences in biomarker concentrations as percentage (%) of the geometric means (Ascorbic Acid: 35-44 years: 4266 mg/L, 65-75 years: 4.290 mg/L; Lutein; 35-44 years: 0.254 µmol/l; 65-75 years: 0.277 µmol/l; α-Carotene: 35-44 years: 0.154 µmol/l; 65-75 years: 0.123 µmol/l; β-Cryptoxanthin: 35-44 years: 0.168 µmol/l; 65-75 years: 0.219 µmol/l; Lycopene: 35-44 years: 0.809 µmol/l; 65-75 years: 0.540 µmol/l; γ-Tocopherol: 35-44 years: 1.126 µmol/l; 65-75 years: 1.350 µmol/l; γ-Tocopherol/Cholesterol: 35-44 years: 0.217 µmol/mmol; 65-75 years: 0.255 µmol/mmol
